# Supplementary material for: Deep learning predicts short non-coding RNA functions from only raw sequence data
Source: PLoS Comput Biol. 2020 Nov 11;16(11):e1008415. doi: 10.1371/journal.pcbi.1008415 (PMC7682815; doi:10.1371/journal.pcbi.1008415)
Supplement: Attachment — Submitted filename: response.pdf [file pcbi.1008415.s003.pdf]

Reviewer #1:

This paper proposes a deep learning method to predict the classes of some non-coding RNAs. It is a little misleading to say “Deep learning predicts non-coding RNA functions”. Besides the well-known classes of non-coding RNAs, the proposed method may not be used to predict the functions of most non-coding RNAs such as lncRNAs, circRNAs, etc.

> **Reply:** Actually functions of long non-coding RNAs are very poorly annotated so there are no, sufficiently trustable, gold-standard that let us estimate, with sufficient confidence, the performance of a supervised classifier. This is true also for circRNAs. So, we agree, the title is a little misleading and we decided to reformulate it as: “Deep learning predicts **short** non-coding RNA functions from only raw sequence data”

It is unclear why the authors “removed sequences greater than 150 bases” as most non-coding RNAs are longer than 200 nucleotides. It is also unclear how sequence redundancy in the dataset was handled.

> **Reply:** This choice was motivated to have an acceptable execution time of all the experiments. To be coherent with the main focus of the revised manuscript (see the previous answer), i.e. short long-non coding RNA, we increased this threshold to 200 to exclude explicitly long non-coding RNAs. More specifically, following also the suggestions of reviewer #2, we extended the dataset of the study to include other classes of short non-coding RNAs, consistent with the focus of the paper. The dataset includes now 88 short non-coding classes and 306016 sequences, almost triplicating the previous datasets, making the study much more robust and tailored for short non-coding RNA.

While k-mer compositions are widely used for input encoding of sequence, they do not capture all the sequential information. Strictly speaking, the k-mer features are not “raw sequence data”. These k-mer features may work well for some ncRNA classes, but not for others.

> **Reply:** Thanks for the accurate question. However, we are not using k-mer composition as a feature. Maybe this was not clearly stated in the original manuscript. Here k-mers are used to represent the sequence itself to be given as input to the Neural Network, maintaining the sequential order of nucleotides (see Figures 3 and 5 in the paper). Specifically, we do not collapse the sequence information into k-mer histograms, rather we encode every k-mer of the sequence as a binary vector. For example, the sequence
>
> AGCTGATT
>
> Is 1-mer encoded as:
>
> (1000)(0100)(0010)(0001)(0100)(1000)(0001)(0001)
>
> As such, we can say the input of the Neural Network is the “raw” sequence suitably encoded by k-mer.

Also, since RNA sequence determines structure that often underlies function, it is unclear how “this finding poses a question against the dogma of secondary structure being a key determinant of function in RNA”.

> **Reply:** With this claim, we would like to figure out an open question maybe in a provocative way. In literature, it is assumed that RNA sequence determines structure that determines function so function depends basically on sequence through its 2d/3d structure (Tinoco et al. “How RNA folds” Journal of molecular biology, 1999). The question we arise observing our results is: are RNA functions determined exclusively going through its 2d/3d structure?
> We observed that our deep network architecture is able to learn functions from lightweight sequence representations, such as k-mers, without precomputing the 2d structure. This is not a trivial question as in literature 2d/3d structure seems to be pivotal to predict functions (see INFERNAL, EDeN and nRC). Computing the 2d/3d structure, through folding tools such as ViennaRNA and iPknot, is very time

expensive. So avoiding it is of course attractive as we show empirically. In addition to the objective result of saving computational time, a consequence is the question whether 2d/3d structure is strictly necessary to predict function. We just discussed qualitatively such aspects without giving data evidence. It may be plausible that the deep architecture capability to learn abstract features even learns the structure to predict the function but it may also be not. We did not go deeper into this aspect and let the question just open.

The deep learning architecture in this study used convolutional neural networks (CNN). I wonder whether some other deep learning techniques, such as recurrent neural network and word embedding, were also tested for this problem.

Reply: Thanks to the referee for pointing this out. We have tested three bidirectional LSTM recurrent neural network (RNN) architectures with an increasing number of nodes (50,100,150) on the dataset (training set and test set) named as “test13” provided by nRC’s author in *Fiannaca et al., 2017*.

Since RNNs are able to process information as sequential data with no predetermined size limit, we have applied these architectures on the sequences encoded as k-mers and not as space-filling curves as they are not sequences but rather 2-D representations of the data.

Table 5 of the manuscript has been updated with these new results. Here, the tested RNN architectures show performances similar to those of the standard CNN architecture. However, the improved CNN architecture still remains the best approach for the classification task object of this study.

Thanks to these results, we believe that a hybrid approach with both CNN and RNN layer blocks perhaps could improve the performance of short ncRNA class classification tasks. Ideally, a first convolutional layer block could identify short sequence motifs correlated with the biological role of the short ncRNA family, and then a recurrent layer block could learn long-term relationships between inferred functional motifs.

We plan to investigate the complexity of this kind of architecture in future works.

What are “non-functional RNA sequences”? The RNA sequences that do not belong to the considered classes can also be biologically functional.

Reply: In the experiments for rejection capability of the algorithm we refer to “non-functional RNA sequences” as sequences randomly generated by shuffling the initial set and preserving the di-nucleotide composition of each original sequence. In the new version, we have clarified this aspect.

The classifier developed in this study should be compared directly with the previous models, especially the ones using RNA structural features. The results for nRC and RNAGCN in Table 6 were taken from a previous study. It is unclear whether the same datasets and testing strategy were also used in the previous study.

Reply: The results reported in Table 6 referred all to the same dataset (training set and test set) named as “test13” provided by nRC’s author in *Fiannaca et al., 2017.* We have re-applied only EDeN on this dataset since the source code, or an executable version, of RNAGCN is not available.

Reviewer #2:

Summary

In recent years, there has been research evidence that secondary structure is the key factor to know the function of RNA. Some machine learning based methods have been successfully proved to be able to predict RNA function from secondary structure information. At present, there are more or less deficiencies in the existing methods for predicting RNA function on the market, such as BLAST, which has a high false negative rate, GraPPLE, which has a high false positive rate, and INFERNAL, which has a high computational cost. In this case, the author proposes a method based

on the original sequence without calculating the known secondary structure features. The method is more robust to the sequence boundary noise and reduces drastically the computational cost allowing for large data volume annotations. The last two advantages together with fast classification speed are essential for large genome annotation.

Major Comments

In general, the idea of this paper is to find a new way to predict RNA function from the original sequence information instead of the existing methods of predicting RNA function through secondary structure, which is of great significance. However, when using k-mer and space filling curve to represent input, the author can add some improvements to these two existing methods to some extent.

> **Reply:** Thanks for the suggestion, indeed the improvements of these representations can be a non-trivial task, however, we emphasize that the contribution of the paper is to show how raw sequence representation can be enough to improve the state of the art in short RNA function prediction avoiding the computation of secondary structure which could be very time expensive.

Secondly, two uncertainty estimators, information entropy and top difference, were evaluated in the prediction of RNA function. For the two uncertain estimators threshold setting, the author lacks the corresponding information.

> **Reply:** They are usually adopted in literature and have been empirically calibrated. Anyway, we have also reported the ROC curve in Figure 9. We make this clearer in the text. Thanks for this point.

Finally, in assessing RNA function, the author assumes that any further structural coding in the input representation does not help improve performance, which remains to be debated and requires corresponding arguments to prove.

> **Reply:** We compared our approach with EDeN, nRC, and RNAGCN. All of them precompute the 2d structure with tools such as ViennaRNA and iPKnot and extract the set of features adopted by the learning algorithm. We observed that our deep network architecture is able to learn functions without pre-computing 2d structure but directly from the raw input sequence and performs more robustly to boundary noise. See the answer to reviewer #1 for further arguments.

Minor Comments

Picture layout: The graphs and tables in the paper are far apart from the content of the text that concerns them and it seems very inconvenient.

> **Reply:** In the new version, we have revised the figure position according to your suggestion.

Supplementary Notes: (13th line from the bottom, page 4) Sentence “In our experiments we consider k varying from 1 to 3” needs to be supplemented to explain why k varies from 1 to 3 and the effect of K on the experiment.

> **Reply:** In the computational scenario of ncRNA classification, mono, di- and tri-nucleotide patterns have always been considered as important discriminative features. We did not explore the effect of k in our experiment but just considered three levels for k as three different input representations. Varying k from 1 to 3 we gain insight spanning from an atomic to a more high level of molecular composition of the sequence.

Subjective argument: (1st line from the bottom, page 5) The sentence “We set empirically the kernel size to 3 and the number of filters at each i-th layer to 32 * 2i” is too subjective in a sense and the author should make it clear what experience the size of the kernel and the number of filters at each i-th layer are based on.

Reply: In order to choose the best set of parameters for our models that better represent the peculiarities of the functional classification problem, we performed first several hyperparameter optimization experiments (data not shown). Regarding the number of filters, we chose an incrementing number of filters in order to expand the representation in the subsequent layers from the previous one. Regarding the kernel size, a smaller size in general helps to capture local and complex features in the data compared to a larger size that extracts features more general and spreads across the sequence. Moreover, with a smaller kernel size the amount of extracted features will be notable, which can be further useful in later layers.

Reviewer #3:

The article by Noviello et al. is a nice investigation on non-coding RNAs for which functional annotations are beneficial for the biological community. The authors exploited deep learning methods to tackle the challenge and their results shed new light on the structure-function relationship in this class of biomolecules.

The authors also provided all the scripts and documentation to reproduce their work and compared their work to other state-of-the-art methods.

The work is nicely written and logical to follow, I have only minor comments to be addressed:

a general proofreading to get rid of the remaining typos and some grammatical errors, or too wordy sentences

Reply: We have revised the text as suggested. Thanks.

I am not an expert on non-coding RNAs and I was wondering in reading about the dataset curation how the 41 classes have been selected and in general to know more about how the classification of non-coding RNA sequences in classes is done. This might be beneficial also for a broad audience as the one of PLOS COMP BIOL.

Reply: The database is almost an updated version of the dataset adopted in Navarin and Costa (Bioinformatics, 2017) which is derived from the RFAM database. To address the issue related to the focus of the paper (reviewer #1) and then to be consistent with the new focus, we decided to further extend the dataset to include almost all short non-coding RFAM classes. The dataset includes now 88 short non-coding classes and 306016 sequences, almost triplicating the previous datasets, making the study stronger. So there is no selection now as all RFAM classes are taken into consideration. To make the focus clearer to a broad audience we added some clarification text in the introduction.

It will be nice if the authors could explain a little bit more the rationale behind the choice of the deep network architecture to this case study instead than other approaches also to benefit a broader audience

Reply: We added some clarification text in the introduction. Thanks.

make the conclusions less technical and more accessible to biologists so that they can really appreciate the value of the work

Reply: We added some clarification text in the conclusion as suggested. Thanks.
